# Artificial Intelligence (AI)-Driven Molar Angulation Measurements to Predict Third Molar Eruption on Panoramic Radiographs

**DOI:** 10.3390/ijerph17103716

**Published:** 2020-05-25

**Authors:** Myrthel Vranckx, Adriaan Van Gerven, Holger Willems, Arne Vandemeulebroucke, André Ferreira Leite, Constantinus Politis, Reinhilde Jacobs

**Affiliations:** 1 OMFS-IMPATH Research Group, Department of Imaging and Pathology, Faculty of Medicine, University of Leuven, and Department of Oral and Maxillofacial Surgery, University Hospitals Leuven, 3000 Leuven, Belgium; arne.vandemeulebroucke@student.kuleuven.be (A.V.); andreleite@unb.br (A.F.L.); constantinus.politis@uzleuven.be (C.P.); reinhilde.jacobs@uzleuven.be (R.J.); 2 Relu, R&D, 3000 Leuven, Belgium; adriaan.vangerven@relu.eu (A.V.G.); holger.willems@relu.eu (H.W.); 3 Department of Dentistry, Faculty of Health Sciences, University of Brasília, Brasília 70.910-900, Brazil; 4 Department of Dental Medicine, Karolinska Institutet, 171 77 Stockholm, Sweden

**Keywords:** artificial intelligence, convolutional neural network, segmentation, orientation, third molar, panoramic radiography

## Abstract

The purpose of the presented Artificial Intelligence (AI)-tool was to automatically segment the mandibular molars on panoramic radiographs and extract the molar orientations in order to predict the third molars’ eruption potential. In total, 838 panoramic radiographs were used for training (*n* = 588) and validation (*n* = 250) of the network. A fully convolutional neural network with ResNet-101 backbone jointly predicted the molar segmentation maps and an estimate of the orientation lines, which was then iteratively refined by regression on the mesial and distal sides of the segmentation contours. Accuracy was quantified as the fraction of correct angulations (with predefined error intervals) compared to human reference measurements. Performance differences between the network and reference measurements were visually assessed using Bland−Altman plots. The quantitative analysis for automatic molar segmentation resulted in mean IoUs approximating 90%. Mean Hausdorff distances were lowest for first and second molars. The network angulation measurements reached accuracies of 79.7% [−2.5°; 2.5°] and 98.1% [−5°; 5°], combined with a clinically significant reduction in user-time of >53%. In conclusion, this study validated a new and unique AI-driven tool for fast, accurate, and consistent automated measurement of molar angulations on panoramic radiographs. Complementing the dental practitioner with accurate AI-tools will facilitate and optimize dental care and synergistically lead to ever-increasing diagnostic accuracies.

## 1. Introduction

The digital revolution in dentistry has largely automated the conventional dental workflow and drastically reshaped the field. Digital innovations have smoothened and accelerated the daily practice and created an important ease-of-use in different areas, resulting in a significant reduction of work time and costs. The introduction of cone-beam computed tomography (CBCT) allowed cross-sectional imaging with limited radiation dose [1]. Consequently, the availability of three-dimensional (3D) imaging data has led to tremendous progress in terms of clinical accuracies and optimization of diagnosis and treatment planning. The use of intraoral scanners and computer-aided systems (computer-aided design/computer-aided manufacturing CAD/CAM) enabled digitized prosthodontics [2,3]. Moreover, 3D-printing is increasingly gaining ground, allowing patient-customized guided surgery [4,5]. Furthermore, recent innovations like virtual and augmented reality created new visualization systems for anatomic exploration [6].

Last decade, also the use of artificial intelligence (AI) progressed remarkably. Deep learning algorithms or convolutional neural networks (CNN) are rapidly emerging in the field of dentomaxillofacial radiology [7]. CNNs are designed to learn patterns from large datasets, without the need for a supervisor labeling the data. The term “deep” refers to the number of (hidden) network layers to progressively extract information and features from the input data. The layers are interconnected via nodes or neurons. Each hidden layer uses the output of the previous layer as its input, thereby increases the complexity and detail of what it is learning from layer to layer [8]. By mimicking human cognition in terms of learning and problem solving, CNNs prove to be as accurate as human observations, and most importantly, allow highly time-efficient and precise calculations [8]. Newly developed AI-tools can assist dentists and dentomaxillofacial radiologists in comprehensive and fast evaluation and documentation of dental radiographs [9]. By synergistically applying CNNs in routine care, we create an outstanding opportunity to optimize our diagnostic capacities and clinical accuracies. CNNs have shown excellent results in diagnosis and classification of diseases, such as caries staging [10], root fracture detection [11], cancer screening [12,13], and diagnosis of periodontal disease [14]. Moreover, AI applications are highly time-saving in preoperative treatment planning in implantology, orthodontics, and orthognathic surgery, by automated detection and segmentation of anatomical structures [15,16]. Furthermore, they allow efficient and precise evaluation of treatment outcomes [17] and can help us towards highly accurate prediction of diseases.

In this context, we developed and validated the first AI-model for automated tooth angulation measurement on panoramic radiographs, in order to predict wisdom tooth eruption potential in adolescent patients. Evaluation of the third molars’ eruption potential at the end of orthodontic treatment allows timely removal of third molars at the stage of lowest risk of mandibular nerve injury [18]. In 1997, Ventä et al. developed transparent device to overlay panoramic images to assess the probability of third molar eruption or impaction [19]. More recently, Begtrup et al. (2013) presented a method for eruption prediction based on cephalometric and panoramic radiographic measurements [20]. Accordingly, panoramic radiographs acquired to evaluate orthodontic alignment at the end of treatment can concomitantly be used to estimate the eruption chances of the third molars. Vranckx et al. (2019) identified a critical third molar angle of 27.0° upward of future functional eruption becomes unlikely [21]. Third molars with an angle greater than 27.0°, relative to the vertical axis of the second molar, tended to enlarge their angulation over time. The purpose of the presented deep learning tool was to automatically extract the orientations of the six molars in the mandible in order to predict the eruption chances of the third molars. The hypothesis was that the tool would be as accurate as human manual angulation measurements, but would show to be highly time-saving in routine workflow.

## 2. Material and Methods

This study was approved by the Ethics Committee of the University Hospitals Leuven and was conducted in compliance with the ICH-GCP principles and the Declaration of Helsinki (2013).

### 2.1. Panoramic Radiographs

Panoramic radiographs were retrospectively selected from the Department of Orthodontics University Hospital Leuven (Belgium) in accordance with the protocol of Vranckx et al. (2019) [21]. Patients with fully erupted mandibular dentition (16 teeth), apart from the third molars, were included. In total, 838 panoramic radiographs were selected: 588 for training and technical validation and 250 for clinical validation of the network. Radiographs were acquired with the Cranex Tome (Soredex, Tuusula, Finland), Veraviewepocs 2D (Morita, Kyoto, Japan) and VistaPano S Ceph (Dürr Dental, Bientigheim-Bissingen, Germany). All image data were anonymized prior to analysis.

### 2.2. Deep Learning Network (CNN) Construction and Training

**Figure 1:**
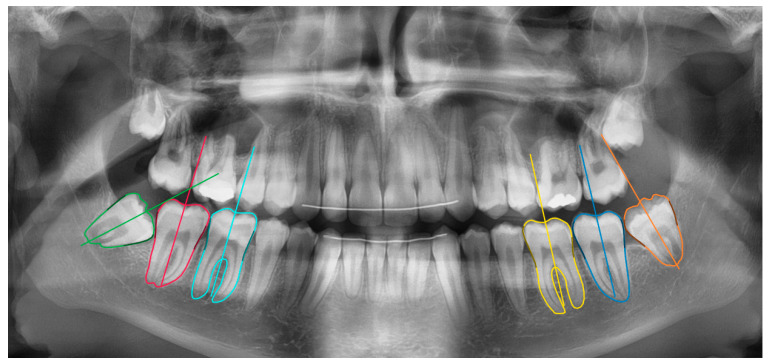
The network calculations were two-fold: six mandibular molar segmentation maps and orientation lines.

The segmentation model was a fully convolutional neural network with pretrained Resnet-101 backbone [22,23]. The network measurements were done in two stages: molar segmentation and orientation estimation (Figure 1). In the first stage, a deep learning algorithm segmented the 6 molars in the mandible. In the second stage, the segmentation maps were used to estimate the molar’s orientation based on an iterative algorithm. These functionalities were integrated inside a software tool based on the open source LabelMe project [24].

#### 2.2.1. Molar Segmentation

The segmentation model was trained and technically validated on a dataset of 550 panoramic images. A separate test set of 38 images was kept for final evaluation. The CNN jointly predicted the molar segmentation maps, and estimations of the middle point of the molars’ occlusal surfaces and the pulp chamber floor. The latter estimated locations served as reference points for an initial estimate of the molar orientations. To reduce overfitting during training, following data augmentations were used: rotations, mirroring, random crops, and changes in illumination (brightness and contrast).

#### 2.2.2. Orientation Estimation

**Figure 2:**
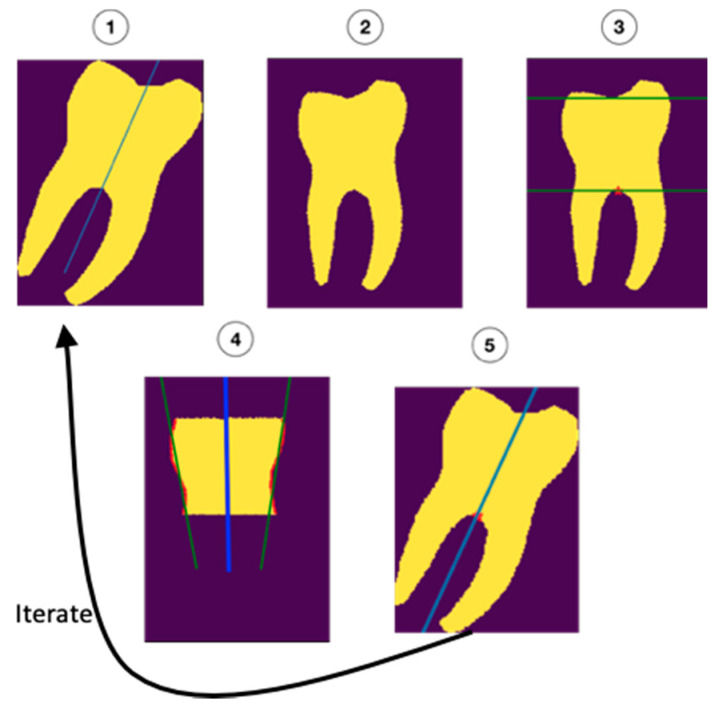
Visual representation of the orientation estimation (iterative algorithm) by the network.

The molar angulations were calculated from the segmentation maps by iteratively refining the initial orientation as described in Figure 2. First, the molars were rotated to the initial (upright) orientation (1 and 2). Next, the roots and occlusal surfaces were censored, leaving a section of the crown and cervix of the tooth which was used to determine the updated orientation (3). Regression was performed on the mesial and distal surfaces (green lines in 4) of this part of the tooth. The average of the orientations of the regression lines (blue line in 4) was added to the initial orientation (5) to form a new, better estimate of the orientation. This process was repeated 10 times to ensure the most accurate fit.

**Figure 3:**
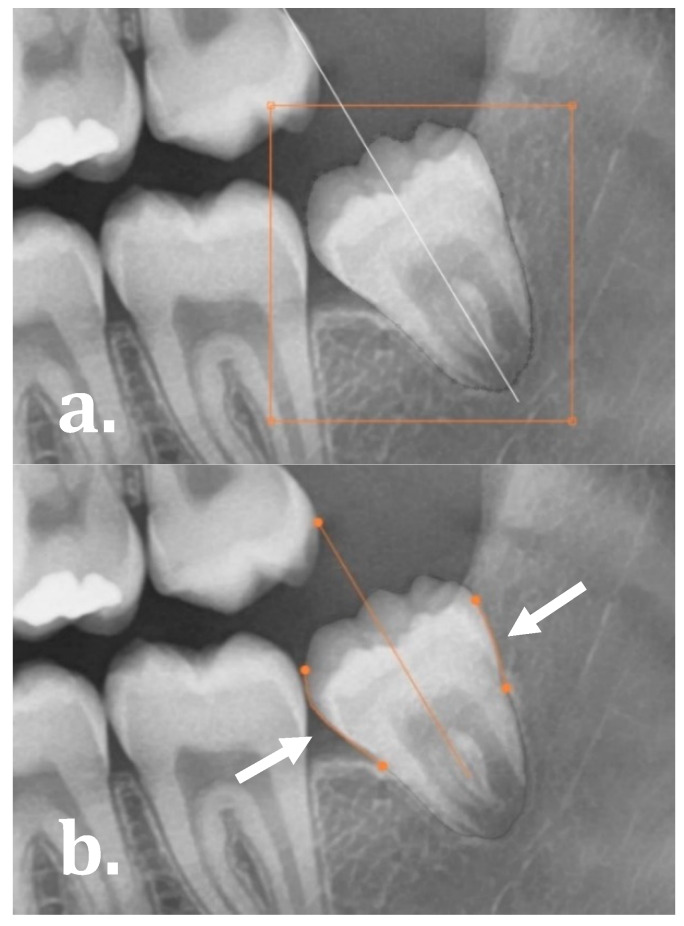
Manual adaptations to the network: (**a**) editing the segmentation map and (**b**) manipulating the orientation line by manually dragging the mesial and distal regression lines.

The software allowed for the user to evaluate and manually refine the final molar angulations by either: (1) editing the segmentation map as demonstrated in Figure 3a (drag, cut or re-create segmentation contours), and (2) manually dragging the start and end points of the regression lines on the mesial and distal side of the molars as illustrated in Figure 3b.

### 2.3. Technical Validation

The deep learning architecture was evaluated based on four accuracy metrics:

- Intersection over Union (IoU): $\frac{TP}{TP+FP+FN}$
- Precision: $\frac{TP}{TP+FP}$
- Recall: $\frac{TP}{TP+FN}$
- Hausdorff distance: the maximal Euclidean distance in pixels between the ground truth and the prediction.

Where *TP*, *FP*, *TN* and *FN* represent the number of pixel-wise True Positives, False Positives, true negative and False Negatives, respectively. An IoU of 1 represents perfect segmentation.

### 2.4. Clinical Validation

To test the clinical validity of our methodology, 250 images with 1500 mandibular molars were measured, of which 500 were third molars. The visual inspection and corrections of the network segmentations and orientations were carried out by two independent observers, both trained to use the software tool at its full extent.

Diagnostic performance of the tool was evaluated based on four parameters. First, the network angulations were compared to the manual measurements from Vranckx et al. (2019) (GIMP 2.0 Ink software, University of California, Berkeley, CA, USA) [21]. Both pre-correction angles (original network calculations) and post-correction angles (final network calculations) were compared to the human reference measurements or clinical reference. Accuracy was quantified as the fraction of correct angulations (with predefined error intervals) compared to the human reference measurements. Error intervals of ±1°, ±2.5° and ±5° were applied. Secondly, the extent of manual refinements or corrections executed on the network calculations was assessed. Both minor segmentation adjustments and total re-creation of the segmentation maps were categorized under manual manipulations. Other parameters considered were tooth position (first M1, second M2, third molar M3, respectively) and development stage of the third molars. This allowed for an intermolar comparison of the segmentation and orientation accuracy. Development stage was recorded based on a shortened version of Demirjian’s classification: follicles without root formation (stages A–D), third molars with starting root bifurcation (stage E) and third molars with root length equal or greater than the crown height (stages F–H) [25]. Lastly, the time consumed for execution of manual measurements vs. automated network calculations, including manual refinements, was recorded for 10% of the sample.

### 2.5. Data Analysis

Performance differences were visually assessed using Bland–Altman graphs, plotting the differences between the manual and AI measurements on the *y*-axis, against the averages of values on the *x*-axis. This statistical method allows to evaluate the agreement between the two methods of measurements (manual vs. AI). Moreover, a Mann–Whitney U test was performed to evaluate the differences between manual refinements to the network executed on M1, M2, and M3. Intraclass correlation coefficients (ICC) were calculated to determine the reliability of angulation measurements among and within observers. The intra-observer agreement also served as a measure of test-retest reliability or consistency of the network calculations. Statistical analyses were performed in MedCalc Statistical Software version 19.2.1 (MedCalc Software Ltd., Ostend, Belgium). The statistical level of significance was set at *p* < 0.05.

## 3. Results

The validation of the sample was based on 250 orthodontic panoramic images of which 109 were males and 141 were females. Mean age was 15 ± 1.9 years old (range 11–21 years). Third molars appeared in three stages of development: 117 early development stage (no roots), 174 starting root formation (bifurcation), and 209 third molars with developed roots.

Table 1 shows the accuracy metrics of the newly developed software tool. The quantitative analysis for automatically segmenting the molars resulted in mean IoUs approximating 90%, which is considered good performance. Considering the lowest mean Hausdorff distances (maximum number of pixels between the clinical reference and AI prediction), M1 and M2 showed the best performance.

### 3.1. Manual Measurements

**Figure 4:**
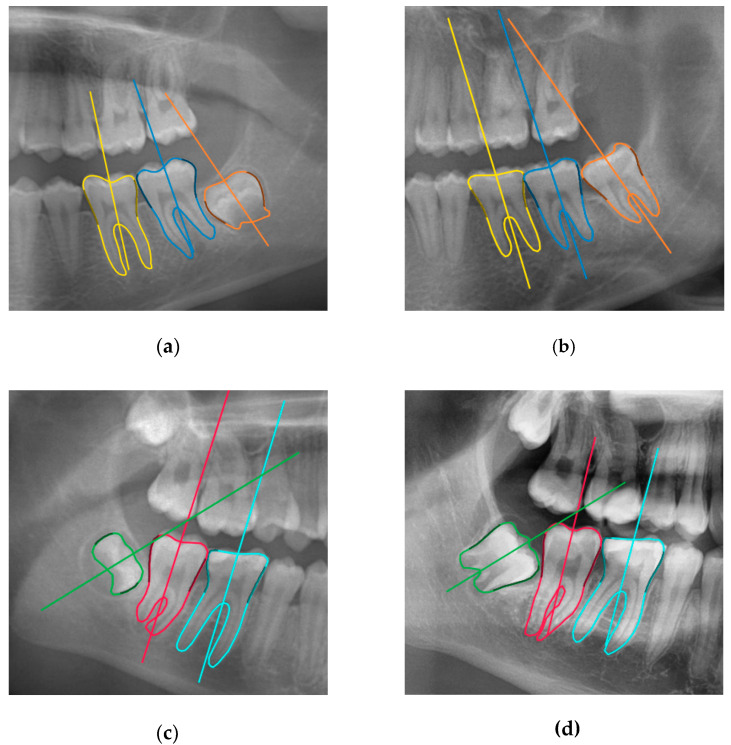
Consecutive panoramic radiographs with a 4-year interval showing third molar eruption in (**a**,**b**); and third molar impaction in (**c**,**d**).

The average manual angle among 1500 measured molars was 27.0° ± 15.0 (range 3.4–75.5°). M1 angles were on average 16.7° ± 5.2, M2 angles 19.4° ± 6.8, and M3 angles 44.8° ± 11.2. When divided into angulation ranges, 1001 (66.7%) molars were angulated in between 0–30°, 459 (30.6%) between 30–60°, and 40 (2.7%) between 60–90°. The data showed 16.4% of the third molars orientated between [24.50°; 29.50°], the acceptable interval around the 27.0° critical angle for eruption, demonstrated by Vranckx et al. (2019) (Figure 4) [21].

### 3.2. Network Measurements

The network results were twofold: the original network angulations and the final network angulations including manual refinements. The average original angle was 28.3° ± 15.6 (range 0.6–88.5°). After minor manual adjustments, the average angle was 28.0° ± 14.7 (range 3.7–77.7°). Table 2 shows the final network angulations closely approximating the manual measurements (for all molars in total and divided per molar).

### 3.3. Network Accuracy

The accuracy of network measurements was calculated using the manual measurements as reference standard. Depending on the applied error intervals of [−1°; 1°], [−2.5°; 2.5°], and [−5°; 5°], the original accuracy of the network was 25.8%, 56.5%, and 83.9%, respectively (Table 3). After minimal manual adjustments to the segmentation maps and/or orientation lines, the final network accuracy increased to 36.6%, 79.7%, and **98.1%,** respectively. The average difference between the manual measurements and the AI calculations was −1.1° ± 1.9 (range −14.1–13.7°). The [−5°; 5°] accuracy was higher in M1 and M2 (99.2%), compared to M3 (96.0%). Right [−5°; 5°] accuracy was slightly better than left (right 98.4% vs. left 97.9%). Excluding underdeveloped third molars (no roots, *n* = 117) from the sample achieved similar network accuracies of 98.3% [−5°; 5°], 79.9% [−2.5°; 2.5°], and 37.3% [−1°; 1°].

### 3.4. Manual Adjustments to the Network Measurements

**Figure 5:**
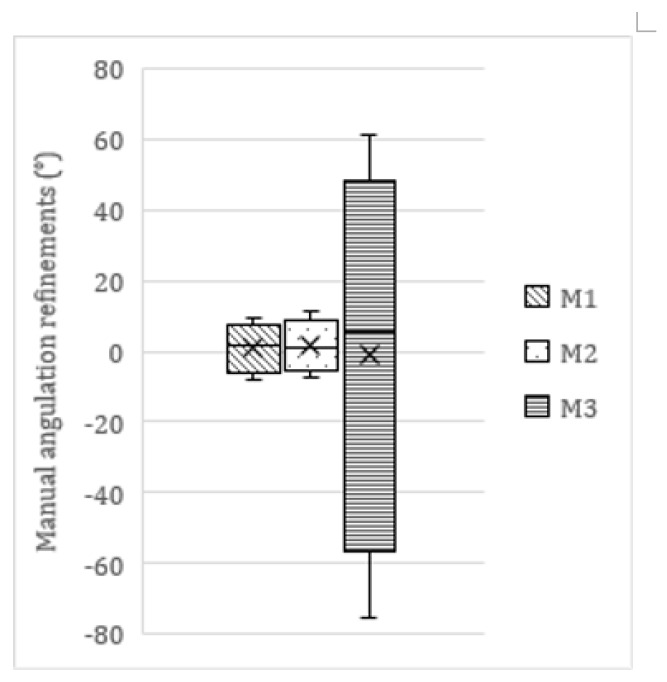
The mean manual refinements on the network measurements displayed per molar. Manual edits to the first (M1) and second (M2) molar were small and limited. Manual edits to the third molar (M3) varied widely. These differences were statistically significant (*p* = *0*.0421).

In 782 molars, minor manual refinements to the network’s segmentation maps and/or orientation lines were necessary. Within this subsample, 64 (8.2%) adjustments fell within the [−1°; 1°] range, 338 (43.2%) within the [−2.5°; 2.5°] range, and 605 (77.4%) within the [−5°; 5°] range. In total, 177 edits were |>5°| (22.6%), of which 72 were |>10°| (9.2%). The average manual refinement was 0.5° ± 6.3 (range −75.5–61.1°). This average refinement differed among molars as displayed in Table 4 and Figure 5. A Mann–Whitney U test showed significant differences in the size/extent of manual refinements executed on M1 and M2, compared to M3 (*p* = *0*.0421).

Almost half of the molars did not require any manual refinement (718/1500; 47.9%). These were mainly molars is the 0–30° range (501/718; 69.8%). Large edits (|>5°|) were mainly performed in molars categorized in the 30–60° range (128/177; 72.3%).

### 3.5. Time Efficiency

**Figure 6:**
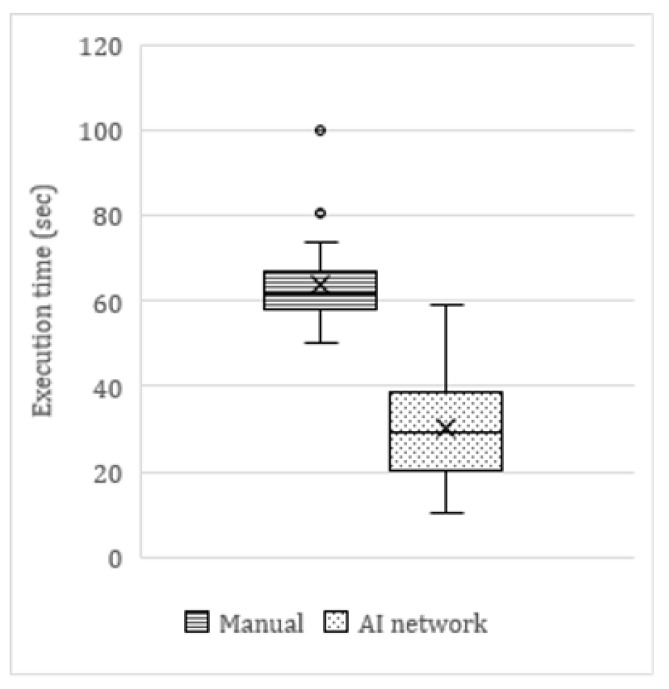
Boxplots showing the time consumed for execution of manual measurements vs. the Artificial Intelligence (AI) network measurements. The network measurements were twice as fast as the manual measurements. The time dispersion for AI measurements was larger, due to some mis-segmentations that needed to be recreated manually.

On average, the AI tool was twice as fast as manual angulation measurements (Figure 6). The average time to manually measure the angulations of six mandibular molars on a panoramic radiograph was 63.9 s, compared to 30.4 s for the AI network (including manual refinements by the observer). This translated as a clinically significant time reduction of 53%. In cases where no manual refinements were needed, the AI network was up to four times faster (<15 s) than the average manual measurements. The few cases in which the network measurements took longer than manual measurements were cases of major errors in the segmentation maps.

### 3.6. Precision and Consistency

**Figure 7:**
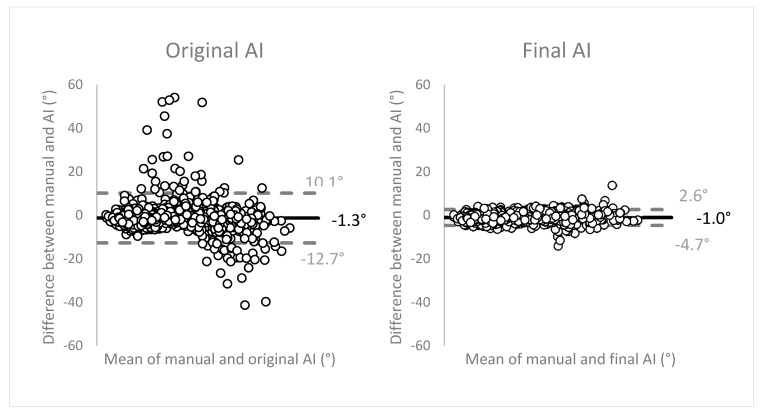
Bland–Altman plots showing good, unbiased agreement between manual and AI molar angulation measurements. Solid line representing the mean, dashed lines representing the upper and lower levels of agreement (mean ± 1.96 SD). Limits of agreement were narrow, translating as high precision of the method of measurement.

Figure 7 displays the Bland-Altman plots to evaluate the agreement between the two methods of measurements (manual vs. AI). The angulation differences between the two methods were plotted on the *y*-axis, against the means of both methods on the *x*-axis. The plots show good, unbiased agreement between the manual measurements and the network. Limits of agreement (LOA) (mean ± 1.96 SD) were narrow, translating as high precision of the method of measurement. The final network measurements within the LOA showed an average of 27.4° ± 14.4 (range 3.7–77.7°). These observations were distributed as: 67.6% in range 0–30°, 29.4% in 30–60°, and 2.9% in 60–90°. The measurements outside of the LOA were angulations with an average of 40.5° ± 14.9 (range 11.2–65.1°), subdivided as 29.0% in range 0–30°, 63.8% in 30–60°, and 7.2% in 60–90°.

Moreover, interobserver agreement was excellent with an ICC of 0.9799 (95% CI, 0.9778–0.9819). Similar excellent scores were recorded for intraobserver agreement, with an ICC of 0.9990 (95% CI, 0.9984–0.9993). At the same time, this excellent intraobserver agreement can be interpreted as an outstanding test-retest reliability or consistency of the network calculations.

## 4. Discussion

The new AI-driven auto-angulation tool showed accurate and fast orientation estimations for third molar eruption prediction. The network automatically segmented the mandibular molars on (postorthodontic) panoramic radiographs, and measured their angulations in order to estimate the eruption chances of the third molars at adolescent age. This allows optimal treatment timing. The present study was a continuation of an earlier research project, of which we automated the angulation measurements with 80% to 98% accuracy [21].

Third molar eruption prediction relies on the fact that severely angulated third molar barely change angulation over time [19,26]. The minimal pre-eruptional angulation changes observed by Vranckx et al. (2019), in combination with the critical angle for (un)favorable direction of rotation (upright or inclined), resulted in reliable estimation of the eruption chances of the third molars during adolescence [21]. Moreover, the available space between the distal side of the second molar and the anterior border of the ramus should be sufficient to accommodate the third molar [20,26]. Consequently, third molars that will fail to erupt in a functional position in the mouth can be timely removed, at early development stage (no roots) and stage of least risk of mandibular nerve injury [18].

The network calculations were twofold: molar segmentation and orientation estimation. Segmentation of teeth for the purpose of treatment planning or surgery preparation is a time-consuming and operator-dependent process [16]. Automation of this treatment step facilitates the dental practitioners’ daily practice. It is generally observed that multirooted teeth present a higher degree of difficulty in automated AI segmentation tools, compared to single-rooted teeth [16]. Since our application focused on mandibular molars only, we observed a relatively high need for manual segmentation refinement. Still, the time-efficiency of our tool was 2 to 4 times faster than manual orientation measurements. Besides, it is important to note that the network measurements came with automatic segmentation maps and orientation calculations, whereas manual angulation measurements were executed by merely visually drawing the vertical midline of the molar based on two predefined reference points, without performing manual segmentation of each molar. This proves the network to be even more time-efficient than at first sight (>53%). Additionally, the test-retest reliability represented by an excellent intraobserver agreement score (ICC 0.9990 with 95% CI 0.9984–0.9993) shows the outstanding consistency of the network measurements. This observation is substantiated by comparing our results with the inter- and intrareliability scores of the manual measurement in Vranckx et al. (2019), ranging from 0.7227 to 0.9604 [21]. Altogether, the combination of automated segmentation and orientation estimation was considered unique, the first of its kind.

Regarding tooth segmentation, the mean IoU approximated 90% for all molars. This performance was similar to other CNNs reported in literature [16,27,28]. Moreover, it is important to state that the CNNs in literature were designed to detect and segment all teeth on a panoramic radiograph, and were not limited to mandibular molars only [15,16,27,28]. Though, fully accurate tooth segmentation (e.g., for treatment planning purposes) was not the main scope of the presented study. Rather, the segmentation maps served as a first step in the orientation estimation process. Therefore, the segmentation maps sufficed to be fully accurate on the mesial and distal sides of the molars to perform the regression. Lower segmentation performance on the molars’ occlusal surface or root apices did not compromise orientation accuracy in any event.

Panoramic radiographs do not reflect a true representation of the 3D dental arches. Subsequently, molar angulations were subject to distortions, because the X-ray beam in panoramic device is not orthogonal to the arch [29]. Taking this into account, the ±5° error interval was considered more than acceptable for third molar eruption prediction. The inherent 2D deformation of the 3D orofacial bone structures on panoramic radiographs makes the molars show wider and the front teeth more narrow [30]. It is only in the critical third molar angulation range of 25–30°, that a smaller error (|<5°|) would be desirable. Fortunately, only 16% of the third molars in our sample appeared in this region. In these selected cases, larger measurement errors could lead to false estimation of the future rotation direction (upright or inclined) of the third molar in development, which could result in less accurate eruption prediction and treatment decision.

It was initially hypothesized that the network would fail segmentation of third molars in early development stages, the stages mostly observed in patients ending their orthodontic treatment [31]. However, the accuracies were very similar among development stages. Moreover, various CNNs have shown to be accurate in all stages of development and are therefore increasingly applied in forensic age estimation [32,33].

Manual refinements to the network segmentations were mostly needed on occlusal surfaces and root level, although this did generally not affect the final orientation estimation. Manual adjustments to the estimated orientations were often the result of the observer’s interpretation of the 3D anatomical relation of the molars to the neighboring teeth and bony structures. So far, this human analysis of panoramic radiographs, taking the overall clinical picture and treatment needs into account, cannot be mimicked by a CNN.

Figure 5 showed wide variability in third molar refinements compared to first and second molars. The latter showed very consistent calculations. High anatomical variability exists in third molar appearance with regards to crown morphology and size, aberrant orientations, multirooted vs. fused roots, etc. Especially proper visualization and interpretation of buccolingual orientations pose a challenge in 2D panoramic radiography. CBCT is more suited to visualize aberrant orientations and the anatomical relation of structures. The ultimate goal is the development and implementation of similar AI-driven segmentation tools in 3D, as CBCT is the most commonly used imaging modality in the virtual preoperative treatment planning of various dental and maxillofacial procedures. Accurate detection, labeling, and segmentation of anatomical structures on CBCT will always be the first and most challenging step in this process [34,35,36]. CNNs can supplement the clinicians in this very time-consuming and tedious task. Moreover, replacing this operator-dependent task by a deep learning network will avert the interobserver variability of manual segmentations [7].

The Bland–Altman plots to evaluate the agreement between the two methods of measurements showed excellent performance of the network, compared to the manual measurements. The high precision of the network measurements is visually demonstrated by the narrow width of the limits of agreement that encompassed more than 95% of the observations. Differences were observed in the distribution of measurements within the LOA (majority 0–30°) and the observations outside of the LOA (majority 30–60°). Altogether, the network performance was considered excellent.

## 5. Conclusions

In conclusion, this study presented and validated a new AI-driven tool for fast, accurate, and consistent automated measurement of molar angulations on dental panoramic radiographs. The network accurately predicted the molars’ segmentation maps and orientation lines, and could be implemented in standard image viewing software to allow easy and fast prediction of third molar eruption at adolescent age. Complementing the dental practitioner with accurate AI tools will facilitate and optimize routine care and lead to a synergistic ever-increasing diagnostic accuracy.

## Figures and Tables

**Table 1:** Accuracy metrics of the new tool for automated molar segmentation and orientation calculation on panoramic radiographs. IoU = Intersection over Union; M1, M2, and M3 = first, second and third molar, respectively.

Mandibular Molar	IoU	Precision	Recall	Hausdorff
**M1**	0.875	0.939	0.928	18.8
**M2**	0.885	0.946	0.933	18.3
**M3**	0.884	0.941	0.938	20.47
**Overall**	0.880	0.940	0.930	19.2

**Table 2:** Average angulations among 1500 mandibular molars on 250 panoramic images: human reference measurements vs. final network results. M1, M2, and M3 = first, second and third molar, respectively.

Mandibular Molar	Manual Measurements	Final Network Measurements
**M1**	16.7° ± 5.2 (range 3.4–32.2°)	17.9° ± 5.0 (range 4.1–31.0°)
**M2**	19.4° ± 6.8 (range 3.9–39.9°)	20.7° ± 6.5 (range 3.7–38.0°)
**M3**	44.8° ± 11.2 (range 5.2–75.5°)	45.4° ± 11.0 (range (7.8–77.7°)
**All molars**	27.0° ± 15.0 (range 3.4–75.5°)	28.0° ± 14.7 (range 3.7–77.7°)

**Table 3:** Network accuracies, quantified as the fraction of correct angulation measurements (with predefined error intervals) compared to human reference measurements.

Accuracy	Original Network Results	Final Network Results
[−1°; 1°]	25.8%	36.6%
[−2.5°; 2.5°]	56.5%	79.7%
[−5°; 5°]	83.9%	98.1%

**Table 4:** Average manual refinements to the network calculations. M1, M2, and M3 = first, second, and third molar, respectively. Min. = minimum; Max. = maximum.

	M1	M2	M3
**Average**	0.74	0.45	0.18
**SD**	2.20	1.75	10.59
**Min.**	−8.03	−7.70	−75.45
**Max.**	9.25	11.07	61.11
